# Validation of a combined ultrasound and bioluminescence imaging system with magnetic resonance imaging in orthotopic pancreatic murine tumors

**DOI:** 10.1038/s41598-021-03684-z

**Published:** 2022-01-07

**Authors:** Juan D. Rojas, Jordan B. Joiner, Brian Velasco, Kathlyne Jayne B. Bautista, Adam M. Aji, Christopher J. Moore, Nathan J. Beaumont, Yuliya Pylayeva-Gupta, Paul A. Dayton, Ryan C. Gessner, Tomasz J. Czernuszewicz

**Affiliations:** 1 SonoVol, Inc, Durham, NC USA; 2 Division of Pharmacoengineering and Molecular Pharmaceutics, Eshelman School of Pharmacy, University of North Carolina, Chapel Hill, NC USA; 3 Joint Department of Biomedical Engineering, University of North Carolina and North Carolina State University, Chapel Hill, NC USA; 4 Lineberger Comprehensive Cancer Center, University of North Carolina, Chapel Hill, NC USA; 5 Department of Genetics, University of North Carolina, Chapel Hill, NC USA

**Keywords:** Cancer imaging, Biomedical engineering

## Abstract

Preclinical mouse solid tumor models are widely used to evaluate efficacy of novel cancer therapeutics. Recent reports have highlighted the need for utilizing orthotopic implantation to represent clinical disease more accurately, however the deep tissue location of these tumors makes longitudinal assessment challenging without the use of imaging techniques. The purpose of this study was to evaluate the performance of a new multi-modality high-throughput in vivo imaging system that combines bioluminescence imaging (BLI) with robotic, hands-free ultrasound (US) for evaluating orthotopic mouse models. Long utilized in cancer research as independent modalities, we hypothesized that the combination of BLI and US would offer complementary advantages of detection sensitivity and quantification accuracy, while mitigating individual technological weaknesses. Bioluminescent pancreatic tumor cells were injected into the pancreas tail of C57BL/6 mice and imaged weekly with the combination system and magnetic resonance imaging (MRI) to serve as a gold standard. BLI photon flux was quantified to assess tumor activity and distribution, and US and MRI datasets were manually segmented for gross tumor volume. Robotic US and MRI demonstrated a strong agreement (R^2^ = 0.94) for tumor volume measurement. BLI showed a weak overall agreement with MRI (R^2^ = 0.21), however, it offered the greatest sensitivity to detecting the presence of tumors. We conclude that combining BLI with robotic US offers an efficient screening tool for orthotopic tumor models.

## Introduction

Murine tumor models are widely used to study the biology of cancer progression and evaluate the efficacy of novel therapeutics^1^. Historically, subcutaneous models have dominated the research landscape due to the ease of creation, and the ability to monitor growth over time with simple handheld calipers. However, recent analyses have demonstrated that subcutaneous models often fail to accurately recapitulate the organ-specific tumor microenvironment and are less appropriate for studying response to therapy^2^. Given the high failure rates of cancer treatments to reach FDA approval^3^, there is a growing consensus that more advanced and human-relevant murine models, such as orthotopically-implanted models, should be replacing traditional subcutaneous cell-line models^1^. Therefore, new methods to longitudinally monitor tumor growth and metastasis in small animal models are becoming important since deep orthotopic tumors cannot be accurately measured with calipers.

Magnetic resonance imaging (MRI) and positron emission tomography (PET) are widely considered to be the gold standard imaging modalities for deep orthotopic tumor evaluation, exhibiting the highest degree of soft tissue anatomical contrast and molecular sensitivity for deep tissue targets, respectively^4^. However, these modalities incur high operational cost, long acquisition times, limited access from high user demand, and ionizing radiation safety considerations (when using PET). For these reasons, optical imaging technologies, such as bioluminescence imaging (BLI), have become ubiquitous due to their excellent sensitivity, ease of use, low cost, and high-throughput capabilities (up to 10 mice scanned simultaneously)^5,6^. BLI, however, is incapable of resolving internal organs, and may be challenged by signal attenuation for tumors growing inside the body cavity resulting in poor correlation to tumor volume^7,8^. Indeed, a recent review recommended that BLI should always be paired with an appropriate non-BLI assessment method to confirm tumor burden measurements^9^.

Ultrasound (US) imaging is an anatomical modality that has been utilized for deep orthotopic tumor imaging^10^. However, despite US and BLI sharing many similar and complementary advantages (e.g. low cost, non-ionizing, fast acquisition)^11^, the two modalities have traditionally not been paired in a dual-mode platform due to inherent physical differences between the instrumentation form-factors. US requires gel or water coupling and is traditionally deployed as a handheld open-air system acquiring data from above the animal (i.e. “top-down”). Conversely, BLI requires a sealed, light tight enclosure with a non-obstructed path for light propagation between the target and camera. Therefore, dual-mode experiments have thus far required repeated manual repositioning of equipment and/or animal to toggle between US and BLI scanning^12^, which reduces throughput, ease of use, and inter-operator consistency. Recently, a new robotic “bottom-up” US imaging approach was introduced that addresses many of the aforementioned challenges^13^. In this system, mice are placed on an acoustically transmissive membrane and scanned from below in 3D by one or more mechanically actuated transducers. With this approach, US images, in principle, can be acquired without obstructing the path of photons to a BLI camera positioned above the animal, thus substantially improving the practicality of a combined US and BLI system.

In this study, we explore the performance (both detectability and accuracy) of a combined US and BLI system, using MRI as a gold standard. We hypothesize that the combination system will offer better deep-tumor detectability than US-alone (i.e. tumor presence can be detected earlier), and that, once detected, tumor burden can be monitored more accurately with the combination system than BLI-alone. Finally, we expect the combination system to offer higher throughput than MRI with minimal reduction in both detectability and accuracy. These hypotheses are tested in an orthotopic pancreatic tumor model in mice, which exhibits aggressive growth and is characterized by areas of hypoxia and high interstitial pressure^14^.

## Methods

The imaging studies were divided into 2 sub-analyses: Robotic US vs MRI to test the ability of US to accurately measure orthotopic tumor volume, and BLI vs MRI to assess the relationship between tumor radiance and tumor volume. All animal studies were performed in accordance with the United States Public Health Service (PHS) policy on Humane Care and Use of Laboratory Animals, following ethical review by the University of North Carolina at Chapel Hill institutional animal care and use committee (IACUC). The reporting in the manuscript follows the recommendations in the ARRIVE guidelines^15^.

### Cancer model and animal protocols

Tumors were implanted in N = 10 C57BL/6 mice (#027, Charles River Laboratories) by injecting 75,000 Luc-tagged KPC 4662 cells mixed at a 1:1 dilution with Matrigel (#354234) and injected using a 28-gauge needle (50 μL total volume)^16^ in the tail of the pancreas. Mice were anesthetized using 1.5 – 3% isoflurane and the left flank was shaved and sterilized with alcohol. A 1–1.5 cm incision was made in the upper left of the abdomen through the skin and body wall. The spleen was exteriorized using forceps in a lifting motion. Tumor cells were injected into the subcapsulary space in the pancreas just below the spleen. A sterile cotton swab was held over the injection site for approximately 20 s to prevent intraperitoneal leakage. Successful subcapsulary injection was confirmed by the presence of a fluid bleb in the pancreas. The abdominal wall was closed using 6–0 sterile surgical suture (Ethicon, Johnson and Johnson) in an interrupted pattern and the skin was closed using sterile wound clips. The animals were monitored for 3 h after the surgery, and daily after that. The wound clips were removed 7 days after the cell injection.

**Figure 1:**
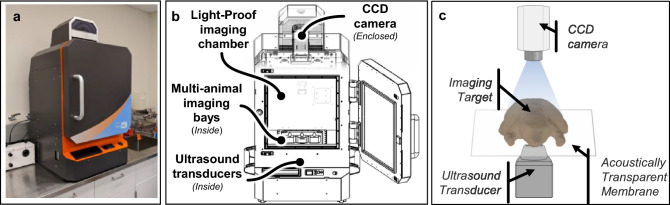
Overview of dual US and BLI imaging system. (**a**) Photo of imaging system. (**b**) Schematic showing the components, including supercooled charge-coupled device (CCD) on top of a light-tight box for BLI and multi-animal bays with anesthesia. (**c**) 3D US volumes are acquired by robotically scanning an ultrasound transducer underneath the animal that is resting on an acoustically transmissive membrane.

Robotic US, BLI, and MRI images were acquired once per week with a Strata US-BLI dual imaging system (SonoVol, Inc., Durham, NC) and a BioSpin 9.4 T MRI system (Bruker, Billerica, MA). A schematic of the dual US and BLI imaging system can be seen in Fig. 1. The mice were imaged in two staggered rounds; imaging for round 1 and 2 started 7 and 9 days after cell injection, respectively. US and BLI images were acquired in the morning, and MR images were acquired in the afternoon. Mice were anesthetized using 1.5–3% isoflurane for induction and data acquisition. Fur was removed from the back and abdomen using an electric razor followed by depilation with Nair prior to US and BLI imaging. During imaging, the animal bays were heated to 37 °C to maintain body temperature. Following longitudinal imaging, animals were euthanized when the longest axis of the tumors reached 1.5 cm, as determined by the veterinary staff.

### Ultrasound imaging

As shown in Fig. 1, mice were placed flat on an acoustically transmissible membrane (proprietary material, SonoVol, Inc.) and a thin layer of water was used to couple the membrane to the tissue. Water was used instead of US gel because it is easier to clean, keeps free of air bubbles, and couples the skin to the membrane more efficiently around surgery scars and wrinkles in the skin. The mice were flat on the membrane and were imaged in the prone and supine positions using a linear array transducer pulsing at 24 MHz. The transducer was pointing up and in contact with the imaging membrane and was robotically scanned to acquire 3D wide-field scans^13^ with a step size of 0.1 mm. Acquisition lasted less than 3 min per mouse (with an additional 2–3 min of prep time for depilation) and consisted of multiple sweeps over a predefined region-of-interest (ROI) that included the entire abdomen.

### Bioluminescence imaging

BLI scans were taken with an integrated CCD camera (Andor, Belfast, UK) supercooled down to − 80 °C, with a 25 mm focal length lens (Navitar, Rochester, NY), as shown in Fig. 1. The camera pointed straight down and was focused 10 mm above the imaging membrane. The F-number was kept at *f*/0.95 throughout the study. Prior to imaging, 250 µL of D-Luciferin (15 mg/mL, XenoLight, Perkin Elmer) was injected intraperitoneally. With animals in the right lateral recumbent position, BLI images were acquired at 15, 20, and 25 min after luciferin injection to ensure the peak signal was captured. An exposure time of 3 min and binning of 4 was used at the beginning of the study, and imaging parameters were updated as the tumors became brighter throughout the study to maximize sensitivity of bioluminescence signal but avoid saturation of the detector.

### Magnetic resonance imaging

Mice were posed in the supine position and the abdomen was scanned using a T2-weighted 2D Turbo-RARE sequence with TR/TE = 3858.12 ms/10 ms, and slice thickness = 0.5 mm. The in-plane resolution was 0.1 mm × 0.1 mm, and 60 slices were acquired to cover the entire mouse abdomen. Breath-gating was used for the acquisitions. Scan and prep time were around 30 and 15 min, respectively, with a total time of 45 min per animal.

### Image analysis

All image analysis was performed using SonoEQ v1.12 (SonoVol, Inc., Durham, NC). US images were manually segmented to obtain tumor volume by six different readers with varying levels of experience in rodent imaging, image analysis, and mouse anatomy. SonoEQ is built on 3D Slicer^17^ (www.slicer.org), an open-source medical imaging analysis platform that allows visualization of 3D volumes by displaying individual 2D slices along each anatomical orientation (Supplementary Fig. 1). To create 3D segmentations, readers utilized either slice-painting-with-interpolation (“Fill Between Slices”) or fiducial-driven 3D model-creation tools (“Surface Cut”) implemented on the 2D slices. The most naïve reader (reader #1) had limited US and mouse anatomy experience and no experience analyzing 3D robotic US images. The most experienced readers (readers #5 and #6) had 3 + years of experience with US imaging, mouse anatomy, and image acquisition and analysis with 3D robotic US. Five of the readers were blinded to the MRI results (readers #1—5) while analyzing images. The sixth reader (reader #6) analyzed the MRI scans, which served as the gold standard for tumor volume measurements. Reader #6 also segmented BLI images in 2D to obtain total flux (photons/s), which is a measure of tumor luminescence.

Prior to segmenting data, readers #1–5 were given approximately 2–3 h of in-person instruction by reader #6. Readers were instructed in mouse anatomy, reading robotic ultrasound, and how to recognize and segment a tumor based on its anatomical location and appearance. Similar to what is depicted in Supplementary Fig. 1, readers were shown full 3D robotic US volumes from a pilot study, which used the same type of tumor model, and were taught to find anatomical “landmarks” such as the kidneys, spleen, and spine, to identify where the tumors would most likely appear and what organs/features would be adjacent. Furthermore, readers were instructed to look for the fluid-filled tumor cores that were sometimes present (Supplementary Fig. 2) and the border around the tumor, which typically looked darker than the surrounding tissue. Lastly, readers were given a practice set of 10 images from the pilot study for segmentation and given feedback on their segmentations prior to analyzing data for this study. Randomization of images was not employed during this study, and readers were allowed to segment images in any order they preferred.

### Growth rate calculation

To calculate the growth rate of the tumors based on the US and MRI measurements, the longitudinal volume values for each mouse (volume vs. time) were fitted to an exponential model defined by the equation $Volume= {Ae}^{\beta t}$, were $A$ is the scalar offset and β is the growth rate^18^. This was performed using the Curve Fitting Toolbox within MATLAB 2020a (Mathworks, Natick, MA).

### Statistical analyses

The US and MRI segmentations from reader #5 and #6, respectively, were used to assess the correlation and agreement between the two imaging modalities using Ran Klein’s Bland–Altman and Correlation Plot toolbox^19^ version 1.10 in MATLAB 2020a and Microsoft Excel. Correlation metrics included Pearson correlation coefficient (*r*), coefficient of determination (*R*^2^), Spearman correlation coefficient (*ρ*) and line of best fit equation (least squares). Additionally, the coefficient of variation (*CV*) and limits of agreement (*LOA*) were also computed. Inter- and intra-reader variability for the US segmentations was measured by computing the intra-class correlation coefficient (ICC) using the degree of absolute agreement definition (“ICC [A-1]”)^20^. Inter-reader variability was measured by computing ICC across all readers. Intra-reader variability was measured from a test–retest experiment performed by reader #6, who segmented the US dataset twice with a separation interval between analyses of 19 weeks. To assess the relationship between anatomical tumor volume and bioluminescence, the *R*^2^ of the tumor volume (US) and total flux (BLI) was calculated. To assess whether the growth rates measured by US and MRI were different, a Mann–Whitney U-test was used to test significance, defined as *p* < 0.05.

## Results

**Figure 2:**
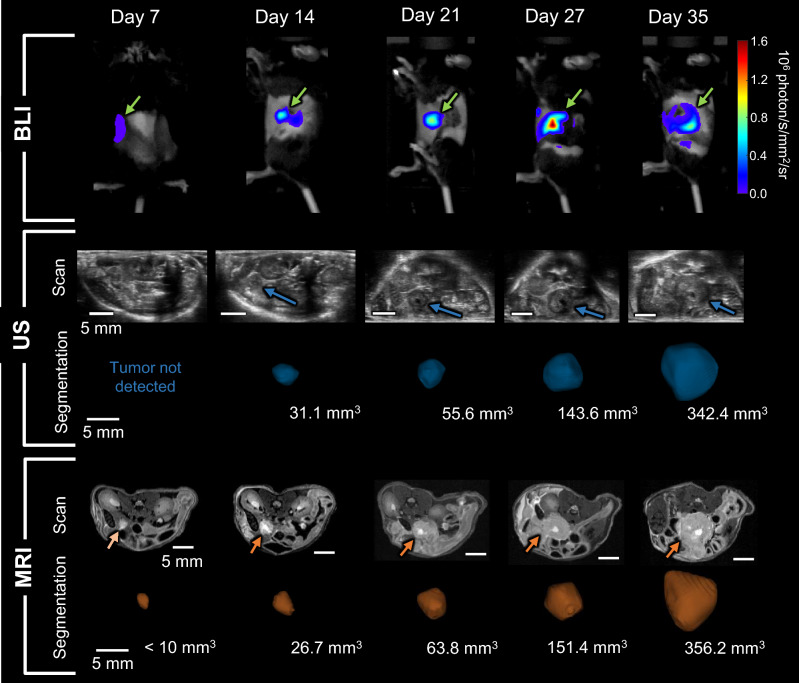
Representative BLI, US, and MRI images through time. BLI, US, and MRI images for 5 imaging timepoints are shown in the first, second, and fourth rows, respectively. Rows 3 and 5 show the 3D rendering of the segmented tumor and its measured volume for each modality. Green (BLI), blue (US), and orange (MRI) arrows point to the tumors. Accurate volume measurement was not possible on the first timepoint. US and MRI images were rotated to show the mouse in the prone position and consist of an axial slice through the volume.

Figure 2 shows representative BLI, US, and MRI images over time, as well as 3D renderings of the segmented tumors. For clarity, only axial slices of the US and MRI 3D volumes are shown. The tumor growth over time can be seen in both US and MRI images, as well as the tumor becoming brighter on the BLI images. Further representative images comparing US and MRI are shown in Supplementary Fig. 1, which include all three slice planes (axial, sagittal, coronal), and identification of other organs such as the spleen, kidneys, and liver, for reference. One of the mice in round 2 died after the first imaging session. Additionally, some mice had to be euthanized by the veterinary staff prior to the final timepoint because the tumors were determined to exceed the size limit (diameter ≥ 1.5 cm). Therefore, the total number of multi-modal imaging datasets captured in this study was N = 36 (Table 1).

**Table 1:** Sample size of mice per imaging day based on protocol tumor size endpoints.

	Day 7	Day 9	Day 14	Day 16	Day 21	Day 23	Day 28	Day 30	Day 35	Total
Round 1	5	–	5	–	5	–	5	–	2	22
Round 2	–	5	–	4	–	4	–	1	–	14

### Tumor detectability

With BLI, all tumors were clearly detectable from the first imaging timepoint (day 7), however, not all tumors were detectable by MRI or US until day 9 and 14, respectively. At day 7, MRI detected 4 out of 5 tumors, while US detected 1 out of 5. However, 3 out of the 4 tumors that were detected with MRI could not be accurately segmented; image intensity indicated the presence of the tumors, but borders were too blurred for confident segmentation (Fig. 2). At all other timepoints, both MRI and US detected all tumors.

### Tumor volume (US) vs. tumor volume (MRI)

**Figure 3:**
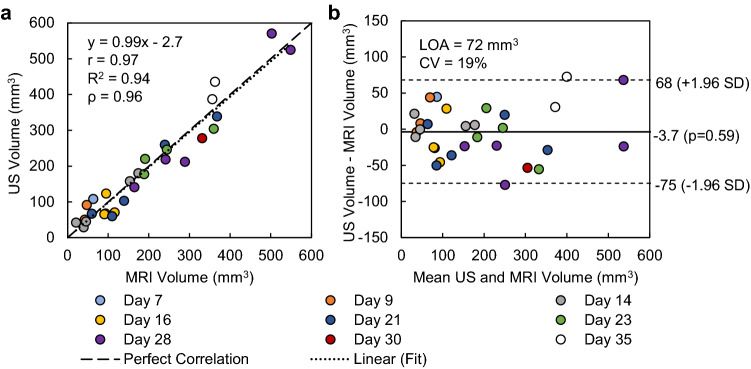
Regression and Bland–Altman plots of US (reader #5) vs MRI volume measurements (reader #6). *r*–Pearson correlation coefficient, *R*^2^–coefficient of determination, *ρ*–Spearman correlation coefficient, *CV*–coefficient of variation, *LOA*–limits of agreement.

**Figure 4:**
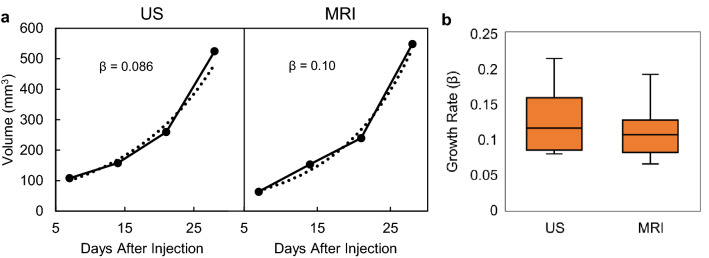
Calculating and comparing tumor volume growth rates. Representative growth curves for the same mouse are shown in (**a**), and box plots of the reported growth rates (β) for all mice as measured by US and MRI in (**b**). The difference in measured growth rates between US and MRI were not significant (p = 0.67).

Figure 3 shows a comparison of US tumor volume segmented by an experienced reader (reader #5) compared against MRI (reader #6). Correlation between the two modalities was strong (*R*^2^ = 0.94), the absolute agreement ICC was high (0.97; 95% CI of 0.94 to 0.99), and the variation in measured tumor volume was small (average absolute difference = 29.4 mm^3^; *LOA* = 72 mm^3^; *CV* = 19%). Bland–Altman analysis also showed that there was no significant bias with robotic US compared to MRI (p = 0.59). The growth rate β was calculated for the US volume measurements of reader #5 and MRI volume measurements of reader #6 (Fig. 4). Example growth curves are shown in Fig. 4, and differences in reported growth rate between US and MRI were not significant (p = 0.67).

### Brightness (BLI) vs. tumor volume (MRI)

**Figure 5:**
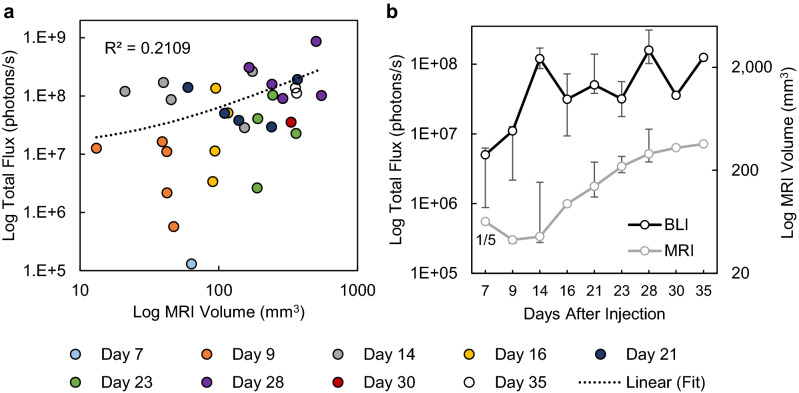
Tumor volume (MRI) and brightness (BLI) comparison. (**a**) Regression plot of tumor volume and total flux obtained from MRI and BLI, respectively. (**b**) The median (± first and third quartiles) tumor volume and total flux over time. *R*^*2*^–coefficient of determination. For improved data visualization, the axes for total flux and MRI volume are both displayed on a log_10_ scale, which makes the linear fit in (**a**) appear curved. The number of tumors that were detectable and large enough to segment is indicated under the marker for the first timepoint (all tumors were detected and segmented after) for the MRI curve in (**b**).

The correlation between photon flux (BLI) and ground-truth tumor volume (MRI) was low, *R*^2^ = 0.21 (Fig. 5). Total flux generally increased over time but had large variability making the median value plateau two weeks after injection, while the measured median tumor volume increased throughout the study. Figure 5b has one volume measurement on day 7 because only a single tumor was large enough to be segmented accurately, while all the tumors were clearly detectable and measurable with BLI on that imaging timepoint. The relationship between BLI and US is not shown here because it is very similar due to the strong correlation of US and MRI, but it is presented in Supplementary Fig. 3.

### Variability between readers

**Figure 6:**
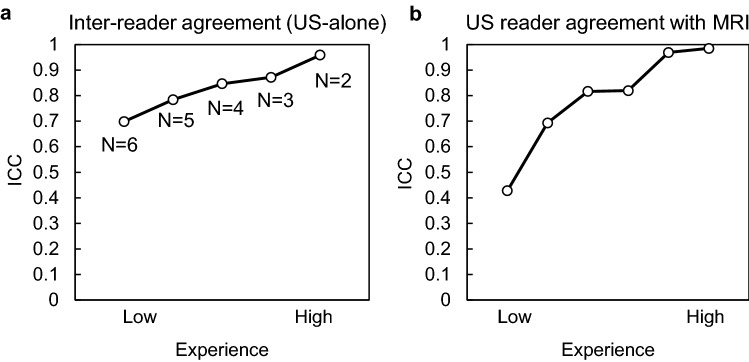
Inter-reader variability and accuracy. (**a**) Plot showing ICC values between US measurements for various groupings of readers based on experience levels. (**b**) ICC values between each individual reader’s US measurements and MRI ordered by experience. *ICC*–intra-class correlation coefficient.

Inter-reader agreement and the effect of reader experience for measuring tumor volume with US is summarized in Fig. 6. The ICC between all six readers was 0.68 (95% CI of 0.52 to 0.82). The variability between readers as a function of experience was assessed by successively removing the least experienced reader and recalculating the ICC, thereby increasing the overall experience of the reader pool with each step (Fig. 6a). The ICC value increased with experience, reaching a value of 0.85 (95% CI of 0.73 to 0.92), when including only the three most experienced readers. Additionally, the agreement between each reader’s US measurements and gold-standard MRI measurements increased with reader experience, ranging from 0.43 for the least experienced reader to 0.98 for the most experienced (Fig. 6b). Finally, the intra-reader agreement (calculated from two successive reading sessions of US images by reader #6) produced an absolute agreement ICC of 0.96 (95% CI of 0.92 to 0.98).

## Discussion

The goal of this work was to evaluate whether a combined US and BLI system could be utilized for improved high-throughput tumor burden assessment of deep-tissue orthotopic tumor models. Our results demonstrate that this two-pronged approach does indeed offer advantages and can achieve comparable performance in terms of tumor detectability and volume accuracy to that of high-Tesla MRI. As expected, BLI was extremely sensitive in detecting the presence of a tumor, exceeding that of both US and MRI, however, it failed to correlate strongly with tumor volume for the chosen orthotopic model, especially at later timepoints (similar to previously published studies^7^). US, on the other hand, was less effective at early tumor detection, however, once tumors exceeded a certain size, the volume could be reliably segmented (Fig. 3) and growth rate measured over time (Fig. 4). These results reinforce the potency of multi-modality imaging, where weaknesses can be mitigated by complementary technologies – BLI can be used as an initial screen to identify tumor hotspots, and then US can add anatomical measurement with soft tissue contrast. Importantly, both robotic US and BLI offer rapid throughput with acquisitions lasting ~ 3 min per animal or per frame, respectively. It has been reported that stand-alone BLI systems can achieve a tenfold reduction in cost compared to MRI due to the faster acquisition times^8^. A tenfold reduction in imaging time may not be achievable when acquiring both BLI and US, but using the dual modality presented here can still be a significant improvement over MRI if US scanning is executed efficiently during luciferin substrate-delivery lag time or by simply acquiring US/BLI one after the other since the acquisition for each modality is about 10 times faster than MRI.

The smallest tumor that was detected in US images by an experienced reader was 21.1 mm^3^ (US: 42.3 mm^3^). However, the agreement between US and MRI for small tumors such as this one (i.e. < 100 mm^3^) was somewhat variable, with absolute differences ranging from 0.2 to 44.0 mm^3^ (Fig. 3b). Some small tumors were segmented with near perfect agreement (MRI: 39.1 mm^3^, US: 34.9 mm^3^), while others of the same volume magnitude were missed entirely (MRI: 42.2 mm^3^, US: not detected). This suggests that the size of the tumor is not the only factor that dictates detectability and segmentation accuracy in 3D robotic US images. In general, US is more susceptible than MRI to artifacts that can give rise to errors in interpretation. Acoustic impedance mismatches from intestinal gas or bone can create shadowing, ringdown, clutter, or reflections that degrade image quality^21^, and, therefore, training/experience likely plays a larger role in US image segmentation. Indeed, more naïve readers exhibited larger variability and poorer ICC values compared to ground truth (Fig. 6). These readers tended to misguidedly focus their segmentations on fluid-filled regions of the tumor (i.e. cysts), which exhibited very high contrast from surrounding tissue and were easy to identify, as opposed to the more subtle tumor tissue border (Supplementary Fig. 2). Improved training regimens, higher frequency transducers, and more advanced B-mode post processing techniques (e.g. clutter suppression, edge enhancement) will likely all improve both the limit of detection by US and also the accuracy with which small tumors are measured.

BLI has been used extensively to track tumor progression in vivo. BLI signal intensity has been shown to be a surrogate for tumor burden^22,23^ and volume in subcutaneous tumors^24^. However, other works have shown that BLI may not correlate well with tumor burden^25^ or volume^26^, especially as the tumors get large^27^. The results of this work show that BLI was well-suited for early confirmation of tumor presence, but a poor substitute for tumor volume, as tumors with the same volume had very different total flux values in this model (Fig. 5). A possible contributing factor for this discrepancy is that some tumors grew with a cystic fluid region (Supplementary Fig. 2), so tumors with the same volume may have different numbers of cells emitting light. However, the correlation of volume (US) and total flux for tumors without a cyst was even lower (*R*^2^ = 0.046), indicating that the presence of a cyst does not explain why BLI was not a good surrogate for tumor volume. There is evidence that BLI does not correlate well with volume when tumors become large due to reduced blood flow to the tumor’s interior altering both luciferin delivery and reaction kinetics^28^. Furthermore, as tumors grow large, the propagation distance to the skin’s surface increases, which can lead to greater photon scatter and attenuation, particularly in orthotopic models where organs can also block the light path. These factors likely contributed to the discrepancy between tumor volume and photon flux, illustrating the importance of having a secondary non-optical modality.

Several limitations were noted in this study. First, to obtain a wide range of tumor sizes, animals were split into two cohorts and imaged on non-overlapping days. Due to this study design, the grouped sample sizes for a given imaging day were small and led to wide error bars when considering tumor volume or flux over time (Fig. 5). A larger cohort might reduce the variability and change the average trends observed over time. Secondly, although readers did complete a training regimen prior to segmenting 3D US images, the number of practice images provided was low (N = 10) when considering the broad range of expertise present in the reader cohort. This limitation was primarily due to the lack of a robust training dataset and will be improved in the future as more animals are scanned with the combination system. Thirdly, BLI and US images could not be overlaid in this study due to the inherent dimensionality difference of the datasets; BLI was captured as a 2D surface radiance while US was captured as a 3D tomographic volume. In the future, we will expand the BLI functionality to include 3D tomographic reconstruction^29^ to enable true co-registration of the two modalities and overlaid anatomical/molecular visualizations (akin to MRI/PET). Fourthly, skin pigmentation has been shown to change in longitudinal BLI studies and affect results when using C57BL/6 mice^30^. In this work, the individual BLI brightness increased for the duration of the study for individual mice, suggesting that pigmentation changes in the skin did not affect the results significantly. However, patterns or trends in the data may have been obfuscated since darkening of the skin was not considered when analyzing the data here, so it should be considered for future work. Lastly, contrast-enhanced US and MRI was not utilized in this study but has previously been shown to help delineate tumor borders and areas of necrosis^31^, and would have likely increased the performance of the less experienced readers. The results here show that strong correlation between US and MRI is possible without contrast agents, but the effect of contrast imaging on demarcating perfused and avascular regions should be explored in the future.

## Conclusion

This work demonstrated a new combination small animal imaging platform for preclinical oncology research and drug development specifically designed for longitudinal assessment of non-superficial orthotopic tumor models. The combination system offers similar advantages of speed and cost as traditional standalone BLI systems but adds a powerful anatomical modality (ultrasound) that previously was too cumbersome to be used in concert with BLI through unique robotic design. Volume measurements of orthotopic pancreatic tumors from robotic ultrasound were shown to correlate strongly with MRI, though readers need sufficient training to achieve accurate readings. Furthermore, as has been described in the literature, BLI was shown to detect tumors earlier than both anatomical modalities (US and MRI), however total flux and volume measurements did not correlate well in this tumor model, illustrating the importance of pairing an anatomical modality to better understand the progression of the disease. As more researchers adopt advanced orthotopic models, combination instruments, such as this one, that enable multiple readouts and mitigate limitations of individual modalities will become increasingly important to accelerate the pace of discovery.

## Supplementary Information

Supplementary Information.
