# Supplementary material for: Validation of a combined ultrasound and bioluminescence imaging system with magnetic resonance imaging in orthotopic pancreatic murine tumors
Source: Sci Rep. 2022 Jan 7;12:102. doi: 10.1038/s41598-021-03684-z (PMC8741958; doi:10.1038/s41598-021-03684-z)
Supplement: Supplementary file 1 — Supplementary Information. [file 41598_2021_3684_MOESM1_ESM.pdf]

Supplementary information

Validation of a combined ultrasound and bioluminescence imaging system with magnetic resonance imaging in orthotopic pancreatic murine tumors

Juan D. Rojas[1], PhD[1]; Jordan B. Joiner, BS[2]; Brian Velasco, BS[3]; Kathlyne B. Bautista, BS[3]; Adam M. Aji, MS[1]; Christopher J. Moore, PhD[1]; Nathan J. Beaumont[1]; Yuliya Pylayeva-Gupta, PhD[4,5]; Paul A. Dayton, PhD[3]; Ryan C. Gessner[1], Tomasz J. Czernuszewicz, PhD[1*]

1. SonoVol, Inc., Durham, NC, USA

2. Division of Pharmacoengineering and Molecular Pharmaceutics, Eshelman School of Pharmacy, University of North Carolina, Chapel Hill, NC, USA

3. Joint Department of Biomedical Engineering, University of North Carolina and North Carolina State University, Chapel Hill, NC, USA

4. Lineberger Comprehensive Cancer Center, University of North Carolina, Chapel Hill, NC, USA

5. Department of Genetics, University of North Carolina, Chapel Hill, NC, USA

*Corresponding author

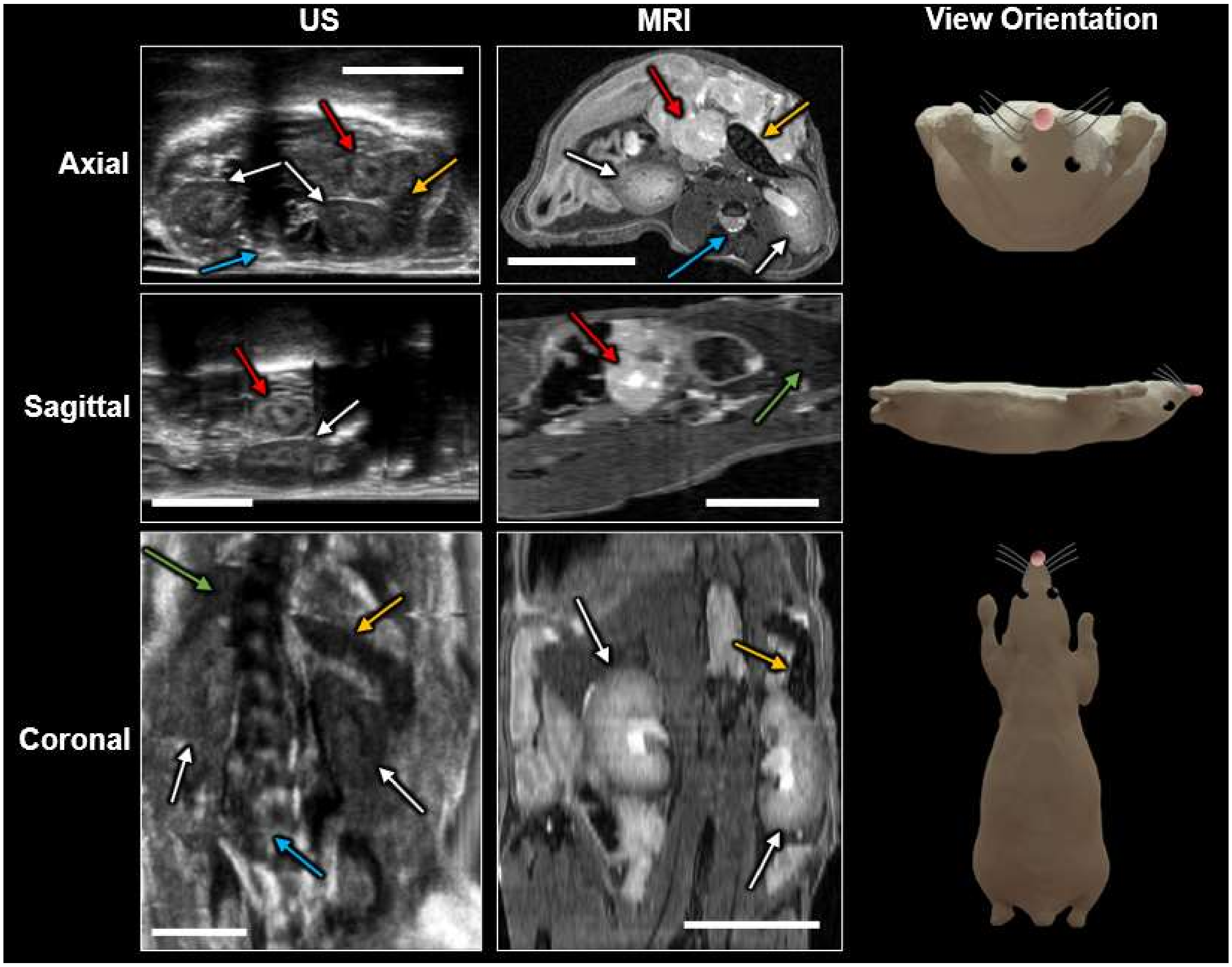

Supplementary Figure 1. Representative US and MRI images. US and MRI images of the same mouse are shown in (a), where the 3D volumes were sliced in 3 anatomical orientations (axial, sagittal, coronal) and displayed as 2D images. Several features such as the spleen (yellow arrows), kidneys (white arrows), liver (green arrows), spine (blue arrows), and tumor (red arrows) can be seen in both sets of images. Scale bars represent 10 mm.

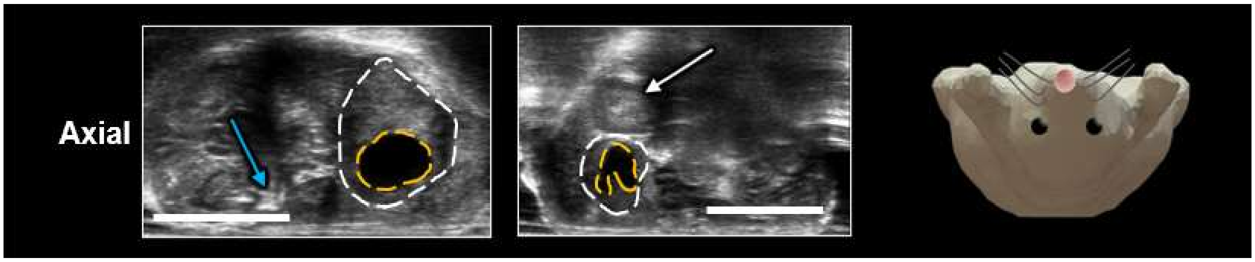

Supplementary Figure 2. Example US images of tumors with hypoechoic cyst. Images show an axial slice of the 3D image. The tumor and cyst are outlined with the white and yellow dashed lines, respectively. Other visible features include the kidney (white arrow) and spine (blue arrow). Scale bars represent 10 mm.

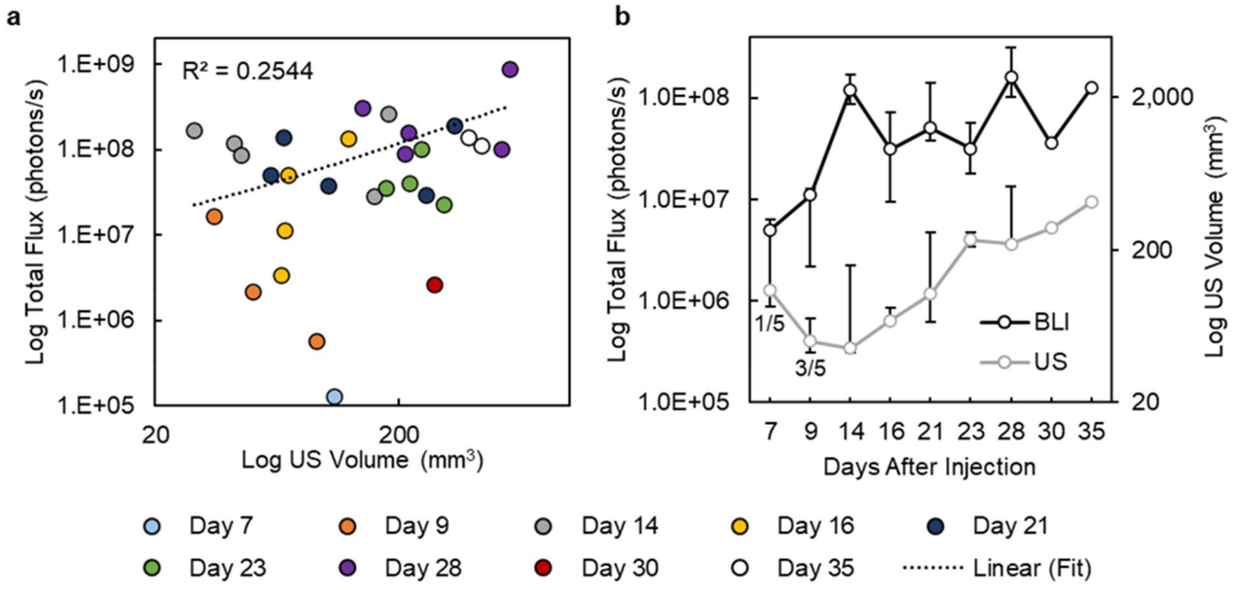

Supplementary Figure 3. Tumor volume (from US) and brightness (from BLI) comparison. (a) Regression plot of tumor volume and total flux obtained from US and BLI, respectively. (b) The median (± first and third quartiles) tumor volume and total flux over time. R^2 -coefficient of determination. For improved data visualization, the axes for total flux and US volume are both displayed on a $\log_{10}$ scale, which makes the linear fit in (a) appear curved. The number of tumors that were detectable and large enough to segment is indicated under the marker for the first 2 timepoints (all tumors were detected and segmented after) for the US curve in (b).

Feature	Ultrasound	MRI (T2)
Tumor	Variable intensity, heterogenous texture, spheroidal shape lacking regular structure	Hyperintense, heterogeneous texture, spheroidal shape lacking regular structure
Tumor border	Dark to bright transition, some speckle texture	Bright to dark transition
Tumor cyst	Very dark, no speckle texture, sharp edge definition	Very bright, no texture, sharp edge definition
Kidney	Variable intensity, dark pelvis, ellipsoidal shape, positioned dorsally to the left and right of spine, regular structure and speckle texture. Can appear visually similar to tumor on first glance due to similar echogenicity and circular shape, but structure is regular, and shape is clearly defined ellipse, while tumors are irregular and heterogeneous.	Hyperintense, very bright pelvis and ureter, ellipsoidal, positioned dorsally to the left and right of spine, regular structure. Clearly identified by geometrical shape.
Spleen	Dark, textured, oblong shape located on left side of animal wrapping around stomach and kidney	Dark, textured, oblong shape located on left side of animal wrapping around stomach and kidney
Stomach/intestines	Amorphous regions with variable intensities on ventral side of animal. Tube-like paths with hyperintense border and dark inner path sometimes visualized if no gas/feces present. Gas/feces appear as hyperintense regions with posterior shadowing.	Hyperintense border with dark inner tube-like paths. Differentiated from tumor by path length and shape.
Spine	Bright specular echo on dorsal side of animal with significant posterior shadowing artifact	Bright regions following the path of cerebrospinal fluid, dark bone cortex

Supplementary Table 1. Appearance comparison of different features in US and MRI (T2) images.
